# A Circadian Clock in the Retina Regulates Rod-Cone Gap Junction Coupling and Neuronal Light Responses *via* Activation of Adenosine A_2A_ Receptors

**DOI:** 10.3389/fncel.2020.605067

**Published:** 2021-01-12

**Authors:** Jiexin Cao, Christophe P. Ribelayga, Stuart C. Mangel

**Affiliations:** Department of Neuroscience, The Ohio State University College of Medicine, Columbus, OH, United States

**Keywords:** circadian rhythm, adenosine, cones, horizontal cells, A_2A_ receptors, rod-cone coupling, gap junction, dopamine D_4_ receptor

## Abstract

Adenosine, a major neuromodulator in the central nervous system (CNS), is involved in a variety of regulatory functions such as the sleep/wake cycle. Because exogenous adenosine displays dark- and night-mimicking effects in the vertebrate retina, we tested the hypothesis that a circadian (24 h) clock in the retina uses adenosine to control neuronal light responses and information processing. Using a variety of techniques in the intact goldfish retina including measurements of adenosine overflow and content, tracer labeling, and electrical recording of the light responses of cone photoreceptor cells and cone horizontal cells (cHCs), which are post-synaptic to cones, we demonstrate that a circadian clock in the retina itself—but not activation of melatonin or dopamine receptors—controls extracellular and intracellular adenosine levels so that they are highest during the subjective night. Moreover, the results show that the clock increases extracellular adenosine at night by enhancing adenosine content so that inward adenosine transport ceases. Also, we report that circadian clock control of endogenous cone adenosine A_2A_ receptor activation increases rod-cone gap junction coupling and rod input to cones and cHCs at night. These results demonstrate that adenosine and A_2A_ receptor activity are controlled by a circadian clock in the retina, and are used by the clock to modulate rod-cone electrical synapses and the sensitivity of cones and cHCs to very dim light stimuli. Moreover, the adenosine system represents a separate circadian-controlled pathway in the retina that is independent of the melatonin/dopamine pathway but which nevertheless acts in concert to enhance the day/night difference in rod-cone coupling.

## Introduction

Due to the rotation of the Earth, ambient (background) illumination gradually changes by ~10-billion-fold over day and night. Retinal function and visual performance depend on the response of the retina to these slow daily changes in illumination and to the actions of an endogenous circadian clock, a type of biological oscillator that has persistent rhythmicity with a period of ~24 h in the absence of external timing cues (Barlow, 2001; Mangel, 2001; Iuvone et al., 2005; Besharse and McMahon, 2016). Although a variety of cellular processes in the retina such as melatonin synthesis and release, dopamine release, and neuronal light responses exhibit circadian rhythmicity, how the retinal clock controls retinal physiology is still not resolved.

**Figure 1:**
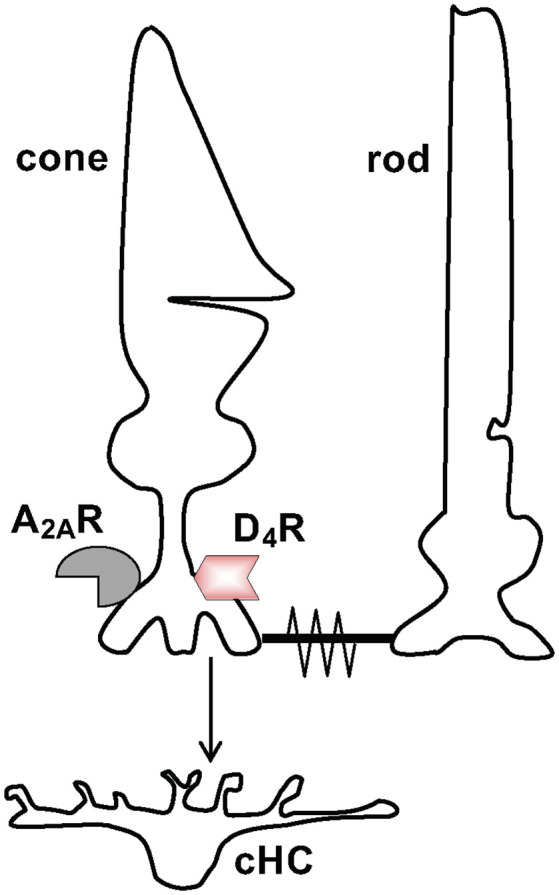
Schematic diagram showing signaling between neurons in the first synaptic layer of the retina. Signaling between cone and cone horizontal cell (cHC) and between cone and rod. Cones release the transmitter glutamate (Glu) to signal cHCs. Also, cones and rods can signal each other *via* rod-cone gap junctions, which are open at night in the dark and closed in the day in the dark and in the light. Both rods and cones express dopamine D4 receptors (D_4_Rs) and adenosine A_2A_Rs although this is illustrated just for cones.

**Figure 2:**
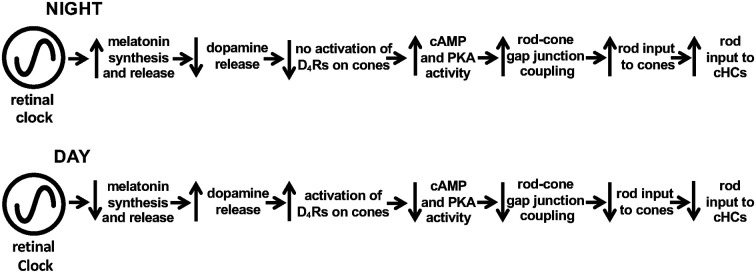
Schematic diagram showing melatonin/dopamine-mediated circadian clock pathway in the fish retina. Previous work has shown that a circadian clock in the retina increases melatonin synthesis and release during the night, which inhibits the release of dopamine from dopaminergic amacrine cells sufficiently so that D_4_Rs on photoreceptor cells are not activated. In contrast, the retinal clock decreases melatonin in the day, which enhances dopamine release, resulting in volume diffusion of dopamine throughout the retina and activation of D_4_Rs on rods and cones. This decreases intracellular cAMP and PKA activity levels in photoreceptors, which lowers the conductance of rod-cone gap junctions so that rod input to cones and cHCs is reduced.

Evidence has shown that the retinal clock utilizes the melatonin/dopamine pathway to adapt to daily changes in ambient illumination (Mangel, 2001; Iuvone et al., 2005; Mangel and Ribelayga, 2010; McMahon et al., 2014; Besharse and McMahon, 2016). Specifically, rod and cone photoreceptor cells synthesize and release the neurohormone melatonin and express dopamine D_4_ receptors (D_4_Rs; see Figure 1), but not D_2_Rs (both of which are members of the D2R family) or dopamine D_1_Rs (Harsanyi and Mangel, 1992; Wang et al., 1997; Witkovsky, 2004; Iuvone et al., 2005). Rods and cones, which are connected by gap junctions (Witkovsky, 2004; Bloomfield and Völgyi, 2009), detect light stimuli, and transmit visual information to post-synaptic neurons (Figure 1). Studies of goldfish, rabbit, and mouse retinas have demonstrated that a circadian clock in the retina modulates rod input to cones and their postsynaptic targets, cone horizontal cells (cHCs), so that very dim rod light signals reach cones and cHCs at night but not in the day (Mangel et al., 1994; Wang and Mangel, 1996; Ribelayga et al., 2008; Ribelayga and Mangel, 2010). The clock achieves this day/night difference by using D_4_Rs to modulate the conductance of rod-cone gap junctions (Figures 1, 2). The clock lowers dopamine release at night so that D_4_Rs on photoreceptors are not activated. This in turn increases intracellular cAMP, which opens rod-cone gap junctions. Conversely, the clock increases dopamine release and D_4_R activation in the day, which decreases intracellular cAMP and closes the gap junctions (Ribelayga et al., 2002, 2004, 2008; Ribelayga and Mangel, 2010; Ribelayga and O’Brien, 2017). The clock-induced opening of rod-cone gap junctions at night allows rods, which directly respond to very dim light stimuli, to transmit this information to cones and cHCs. As shown in Figure 2, increased release of dopamine in the day compared to night results from the circadian rhythm of melatonin synthesis and release, which are greater at night than in the day, and from the inhibitory effect of melatonin on dopamine release (Ribelayga et al., 2004; Witkovsky, 2004; Iuvone et al., 2005; Besharse and McMahon, 2016). However, the robust day-night difference in rod-cone coupling suggests that other clock-controlled effectors may also contribute to the regulation of rod-cone coupling. More specifically, although our lab has shown that the increase in dopamine release and D_4_R activation in the day decreases rod-cone coupling (Ribelayga et al., 2002, 2008), these results do not establish whether the robust increase in rod-cone coupling at night results simply from the lack of D_4_R activation and/or to the action of an additional clock effector.

An additional effector that could contribute to the large day/night difference in rod-cone coupling is the purine adenosine. Adenosine is a natural metabolite that plays numerous roles in the central nervous system (CNS), including the modulation of neuronal activity and neuroprotective actions in response to hypoxia and ischemia (Dunwiddie and Masino, 2001; Latini and Pedata, 2001; Chen, 2014; Cunha, 2016; Ballesteros-Yáñez et al., 2018). Also, adenosine mediates regulation of numerous rhythmic phenomena such as the sleep/wake cycle (Basheer et al., 2004), the modulation of retinohypothalamic input to the suprachiasmatic nucleus (Watanabe et al., 1996), rhythmic gene expression in the pituitary (von Gall et al., 2002), and the production of pineal melatonin (Nikodijevic and Klein, 1989). Adenosine signaling is mediated by four pharmacological/molecular receptor subtypes named A_1_, A_2A_, A_2B_, and A_3_. Activation of A_**1**_Rs or A_3_Rs inhibits adenylate cyclase through Gi/o proteins, whereas activation of A_2A_Rs or A_2B_Rs stimulates adenylate cyclase through Gs proteins (Dunwiddie and Masino, 2001; Chen, 2014; Cunha, 2016). A_**1**_Rs and A_**2A**_Rs are distributed throughout the brain, whereas A_**3**_Rs and A_**2B**_Rs are expressed at low levels (Liu et al., 2019).

Evidence suggests that adenosine is also an important neuromodulator in the vertebrate retina (Blazynski and Perez, 1991; Lohr et al., 2014; Dos Santos-Rodrigues et al., 2015). Retinal cells possess A_1_, A_2A_, A_2B_, and A_3_ receptors and enzymes of the adenosine metabolic pathway (Blazynski and Perez, 1991). Also, adenosine mediates the physiology of many retinal cell types such as cones, which express A_2A_Rs in addition to D_4_Rs (Figure 1; Blazynski, 1990; Kvanta et al., 1997; Li et al., 2013; Lohr et al., 2014; Dos Santos-Rodrigues et al., 2015). For example, adenosine inhibits Ca^2+^ influx into cone synaptic terminals (Stella et al., 2003), suppresses exocytosis from cone terminals (Stella et al., 2009), enhances phosphorylation of photoreceptor gap junction proteins at night (Li et al., 2013, 2014), and stimulates fish cone myoid elongation during the day (Rey and Burnside, 1999). Moreover, endogenous levels of adenosine in the mammalian retina are controlled both by light/dark adaptation and a circadian clock, so that adenosine is highest at night in the dark (Ribelayga and Mangel, 2005).

However, it has not been established whether the circadian clock that regulates retinal adenosine is located within the retina or elsewhere in the CNS. It is also not known whether circadian clock-controlled alterations in adenosine modulate neuronal light responses and information processing in the retina. We, therefore, studied whether adenosine functions as an endogenous effector of the retinal clock by activating cone A_2A_Rs at night. We used a variety of techniques in the intact goldfish retina including measurements of adenosine overflow and content, tracer injections into individual cones to measure the extent of photoreceptor gap junction coupling, and electrical recording of the light responses of cone photoreceptor cells and cHCs. The results show that a circadian clock in the retina itself controls extracellular and intracellular adenosine levels so that they are highest during the subjective night. Moreover, the clock regulates adenosine independently of melatonin and dopamine receptors. Also, we report that circadian clock control of endogenous A_2A_R activation increases rod-cone gap junction coupling and rod input to cones and cHCs at night. These results, together with previous findings concerning the melatonin/dopamine system, suggest that the adenosine system is controlled by a retinal clock(s) independently of the melatonin/dopamine pathway and that endogenous activation of cone D_4_Rs in the day decreases rod-cone gap junction coupling and rod input to cones and cHCs (Figure 2), whereas endogenous activation of cone A_2A_Rs at night increases rod-cone coupling and rod input to cones and cHCs.

## Materials and Methods

### Ethical Approval—Animals

Experiments were performed on retinas obtained from common goldfish (*Carassius auratus*) approximately 15–18 cm in length supplied by Ozark Fisheries, Incopration (Stoutland, MO, USA). The care and use of the fish were in strict accordance with the recommendations in the Guide for the Care and Use of Laboratory Animals of the National Institutes of Health. The protocol, including the method of killing the fish, was approved by the Institutional Laboratory Animal Care and Use Committee of The Ohio State University. After deeply anesthetizing fish with methanesulfonate (MS222, 150 mg.L^−1^), euthanasia was achieved by decapitation followed by double pithing. All the necessary steps were taken to minimize animal suffering. A total of ~240 fish were used for this study. Goldfish were chosen for this study because we can build on previous work on circadian control of rod-cone coupling in goldfish retina and because goldfish cones are relatively large compared to those found in the retinas of other species, including mammals, facilitating the study of these cells with electrophysiological recording techniques.

Fish were maintained at 22 ± 1°C on a 12 h light/12 h dark (L/D) cycle (with lights-ON at 03:00 a.m.) for at least 2 weeks before an experiment. During circadian experiments, fish were kept 24–48 h in darkness before surgery. Throughout this paper, “subjective day” refers to the time of the circadian cycle [circadian time (CT) 0–12] during which illumination was previously present [i.e., projected Zeitgeber daytime (ZT 0–12) from the previous L/D cycle]. Subjective night refers to the time of the circadian cycle (CT 12–24) during which illumination was not previously present [i.e., projected Zeitgeber nighttime (ZT 12–24) from the previous L/D cycle]. As a result, for the subjective day experiments, the fish were kept in darkness from the end of the previous day (lights-off + 15 h). In contrast, for the subjective night experiments, the fish were kept in darkness from the beginning of the previous night (lights-off + 27 h). Subjective day and subjective night experiments were performed at ZT 04–08 and ZT 15–19, respectively.

### Tissue Preparation

Following euthanasia, an eye was enucleated and the intact neural retina isolated, as described previously (Wang and Mangel, 1996; Ribelayga et al., 2002, 2004, 2008; Ribelayga and Mangel, 2003, 2007). All surgical procedures were performed in darkness using infrared goggles (AN/PVS-5; Night Vision Equipment, Emmaus, PA). In some experiments, the retinal pigment epithelium and sclera (RPE-S), minus the neural retina, were studied together (Figure 5B).

**Figure 3:**
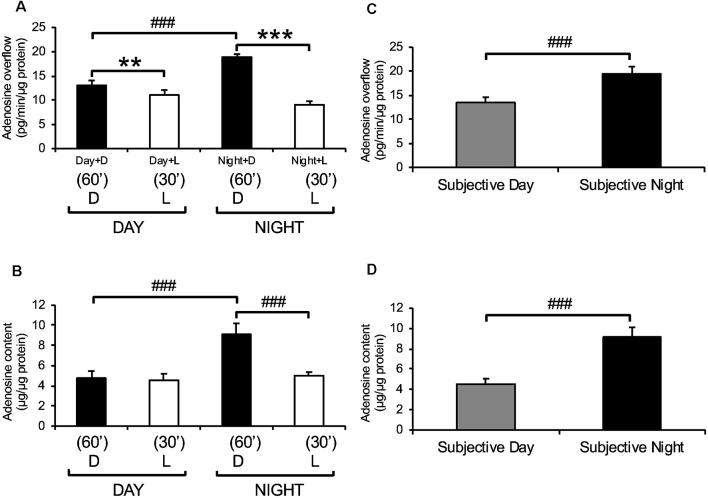
Endogenous adenosine levels in the retina exhibit light/dark and day/night differences. Dark-adapted goldfish neural retinas were isolated in the middle of the day, night, subjective day, and subjective night and superfused in the dark (D) for 1 h at the end of which adenosine was assayed. Thereafter, a white light background (L) in the low photopic range (0.2 mW/cm^2^) was applied for 30 min. **(A)** Adenosine overflow from isolated neural retinas collected during the day and night. **(B)** Adenosine content in isolated neural retinas collected during the day and night. **(C)** Adenosine overflow from isolated neural retinas collected during the subjective day and subjective night. **(D)** Adenosine content in neural retinas isolated during the subjective day and subjective night. Darkness, compared to light stimulation, increased adenosine overflow in the day and night and increased adenosine content at night. However, darkness did not affect adenosine content in the day. In contrast, the circadian clock increased both adenosine overflow and content at night compared to the day. Data are from 5–6 retinas/condition ± SEM. **p < 0.01, ***p < 0.001 (paired Student’s *t*-test); ^###^*p* < 0.001 (unpaired Student’s *t*-test).

**Figure 4:**
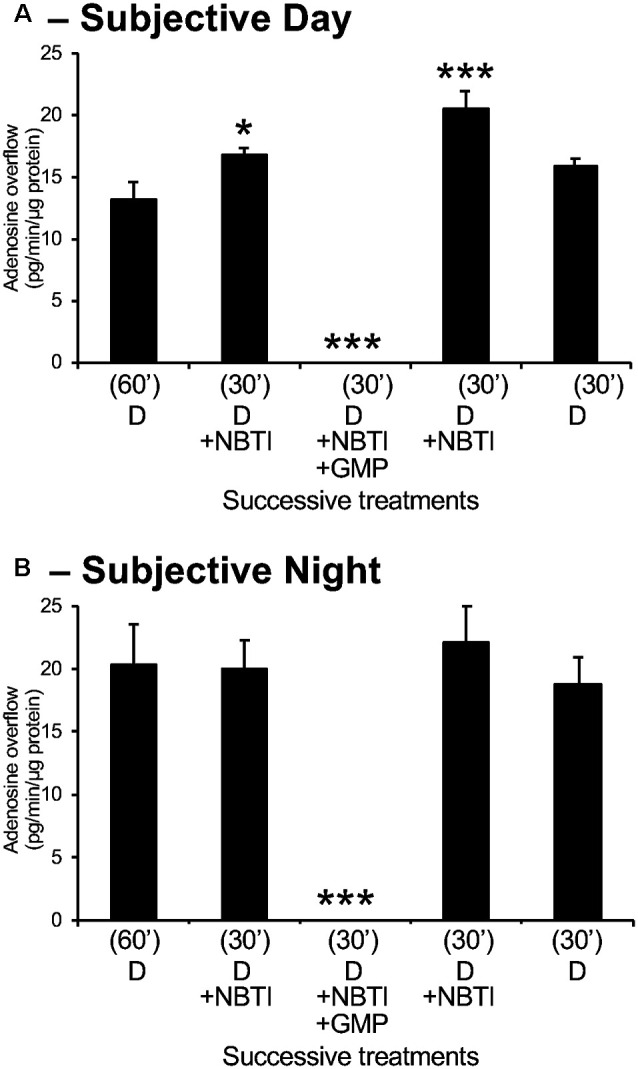
Adenosine measured in retinal overflow is of extracellular origin. Isolated neural retinas were superfused for 60 min after surgery during the day **(A)** and night **(B)**. Thereafter, the retinas were superfused for 30 min with 10 μM of the bidirectional adenosine transport blocker NBTI and subsequently with NBTI+ the ectonucleotidase inhibitor GMP (1 mM). NBTI increased adenosine overflow during the day but did not affect at night. Also, the superfusion of NBTI+ GMP suppressed the extracellular level of adenosine during both the day and night. These observations demonstrate that extracellular adenosine is of extracellular origin. Note that adenosine overflow was higher during the night compared to the day, but the effects of NBTI were more pronounced during the day than at night. Data are from 5–6 retinas/condition ± SEM. *p < 0.05, ***p < 0.001 (Student-Newman-Keuls multiple comparison test following RM-ANOVA).

**Figure 5:**
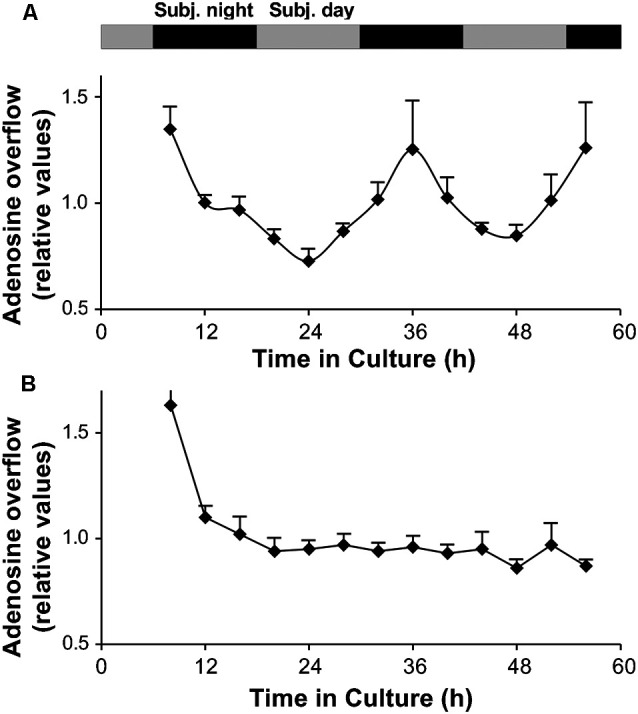
A circadian clock in the goldfish neural retina increases extracellular adenosine at night. **(A,B)** Long-term persistence of the circadian rhythm in adenosine overflow was observed from isolated intact neural retinas **(A)** but not from RPE-S **(B)** kept under constant conditions (see “Materials and Methods” section). Adenosine overflow was measured for more than 2 days at 4 h intervals when the culture medium was changed. The first data point was not included in the graph. Relative adenosine levels (amount of adenosine in each fraction divided by the average) are shown for better comparison between different retinas and between different RPE-S. Extracellular levels of adenosine were rhythmic (RM-ANOVA, *F*_(12,48)_ = 5.852; *P* < 0.0001). COSINOR analysis of the rhythm revealed a period of 25.2 ± 1.6 h (*p* < 0.001, see “Materials and Methods” section for details). Data are from five retinas. **(B)** No circadian variations of adenosine overflow were observed from RPE-S under the same constant dark and temperature conditions (RM-ANOVA, *F*_(12,36)_ = 0.767; *P* = 0.669). Data are from four RPE-S. **(A,B)** Gray and black bars indicate the subjective day and subjective night, respectively. Before stabilizing ~10 h following surgical isolation of tissue, adenosine overflow was relatively high, likely due to an acute effect of surgery. Data collected 4 h after surgery were therefore not included in the analysis of rhythmicity and are not shown.

### Measurement of Endogenous Adenosine

#### Short-term Superfusion Conditions

Intact neural retinas were placed in a custom-made closed superfusion chamber (1 ml). A peristaltic pump delivered the superfusion solution at a constant rate of 0.5 ml/min so that the superfusate inside the chamber was replaced every 2 min. The superfusion solution contained (in mM): 125 NaCl, 25 NaHCO_3_, 10 glucose, 2.5 KCl, 1.0 MgCl_2_, 0.7 CaCl_2_. Experiments were performed at room temperature (22 ± 1°C). In a series of preliminary experiments, we observed that under these conditions adenosine overflow underwent a surge when the superfusion began, but stabilized after 60 min and remained stable thereafter for at least 3 h in darkness. For this reason, experiments always started after an initial 60 min of superfusion in darkness. We also recorded the electroretinogram (ERG) of isolated neural retinas after 1, 2, and 3 h of superfusion. No changes in the amplitude and kinetics of the b-wave and slow PIII (sPIII) throughout superfusion were observed, demonstrating that our preparation was viable and therefore suitable for pharmacological manipulations.

#### Long-term Organotypic Culture Conditions

Intact isolated neural retinas (i.e., minus the RPE-S) and RPE-S without retina were cultured in constant total darkness (< −10 log Io) for 56 h as described previously (Ribelayga et al., 2002, 2004). Briefly, retinas or RPE-S were maintained under a water-saturated 5% CO_2_/balanced with O_2_ atmosphere at 20 ± 0.1°C in 2 ml of a Ringer based-solution that contained the components described above as well as 20 ml/L of 50× MEM amino acids solution (without glutamine), 10 ml/L of a 100× MEM vitamin solution, and antibiotics (100 U/ml penicillin and 100 μg/ml streptomycin). The culture medium was completely changed every 4 h. All supplements were from Atlanta Biologicals (Atlanta, GA, USA).

#### Adenosine Measurements

The adenosine level in the culture medium or superfusate, representing adenosine overflow, and the adenosine level in the homogenate, representing adenosine content, were measured using high precision liquid chromatography (HPLC) with fluorescence detection (Agilent 1100 Series form Agilent Technologies/Hewlett-Packard, Germany), as described previously (Ribelayga and Mangel, 2005). Briefly, adenosine was derivatized or transformed into 1,*N*^6^-etheno-adenosine, a fluorescent compound. Following derivatization, adenosine was extracted using an agarose-based resin (aminophenylboronate agarose, ProMetic Biosciences Limited, UK). Components were separated on an adsorbsphere HS (C18) column (250 mm × 4.6 mm, 5 μm porous silica, Alltech, Deerfield, IL, USA). The column was operated with an isocratic mobile phase composed of 0.05 M NH_4_C_2_H_3_O_2_ (pH 6.0) and 12% methanol (Thermo Fisher Scientific, Pittsburgh, PA, USA). The mobile phase flow rate was set to 0.7 ml/min and the column temperature to 30°C so that the pressure in the column was ~1500 psi. For each run, the amount of adenosine quantified was corrected using tubercidin as an internal standard. The measured values of adenosine were then normalized to the protein content of each retina.

### Electrophysiology

#### Light Stimuli

Responses of cones and cHCs to dim full-field white and spectral light stimuli were recorded. Light stimuli were provided by a 100 W tungsten-halogen lamp. The maximum, unattenuated intensity (*I*_o_) of full-field white light stimuli was 200 μW/cm^2^. Intensity values indicated in the text are relative to *I*_o_. Calibrated neutral density filters and narrow-band interference filters were used to control light intensity and stimulus wavelength, respectively.

#### Intracellular Recordings of Cone Horizontal Cells

Standard intracellular recording procedures were employed to record the light responses of fish L-type (H1) cHCs, as described previously (Ariel et al., 1986; Wang and Mangel, 1996; Wang et al., 1997; Ribelayga and Mangel, 2003). Briefly, intact isolated neural retinas were placed in an open chamber that had a volume of 1 ml and were superfused at 0.5 ml/min with a Ringer’s solution of the same composition as used for short-term superfusion experiments (see above). After surgery, retinas were dark-adapted for at least 1 h, following which horizontal cells were impaled without the aid of any light flashes. cHCs were identified as previously described (Ariel et al., 1986; Wang et al., 1997; Ribelayga et al., 2002, 2004).

#### Patch-Clamp Recordings of Goldfish Cones

Whole-cell patch-clamp recordings (current-clamp configuration with *I* = 0) from the inner segments of individual cones in intact goldfish neural retinas were made under visual control, as described previously (Ribelayga et al., 2008). The pipette solution contained (in mM) 20 KCl, 100 D-K-gluconate, 7.48 KHCO_3_, 5.0 HEPES, 1.0 MgCl_2_, 4.0 Na_2_-ATP, 0.02 Na_3_-GTP, 1.0 CaCl_2_, and 1.0 EGTA. The pH was adjusted to 7.3 with KOH and the solution was divided into aliquots and kept frozen. Neurobiotin (0.3%) was added fresh daily to a sample of pipette solution. Individual cones were labeled by iontophoresis of the biotinylated Neurobiotin tracer (MW = 373) during whole-cell patch-clamp recording by maintaining the recorded cone at +20 mV for 10 min. After an additional 30 min in the dark, retinas were fixed in 4% paraformaldehyde in 0.1 M phosphate buffer (PH 7.4) at room temperature for 1 h. The tissues then were washed and labeled. Fluorescent photoreceptors, indicating the presence of Neurobiotin tracer, were subsequently visualized with streptavidin-conjugated-Alexa 488 (Jackson ImmunoResearch, West Grove, PA, USA). Labeled cells were imaged, photographed, and counted with a Leica SP8 laser scanning confocal microscope (Leica Microsystems Inc, Buffalo Grove, IL, USA). Tracer-coupled rods and cones were counted with NIH ImageJ software (Ribelayga et al., 2008).

#### Test Drugs

The accumulation of extracellular adenosine arises from two different sources, either from the conversion of extracellular ATP into adenosine *via* the sequential actions of an ectoATPase and an ectonucleotidase or from the intracellular conversion of AMP *via* the action of an endonucleotidase (Liu et al., 2019). Clearance from the extracellular space requires reuptake and intracellular breakdown of adenosine by adenosine deaminase or adenosine kinase (Dunwiddie and Masino, 2001; Latini and Pedata, 2001). To determine whether the source of extracellular adenosine was intracellular or extracellular, we used two selective drugs applied alone or in combination, namely the 5′-ectonucleotidase blocker GMP (1 mM; Rosenberg et al., 2000) and the equilibrative adenosine transport blocker *n*-nitrobenzylthioinosine (NBTI, Griffith and Jarvis, 1996). Plasma membrane transport of nucleosides occurs by sodium-dependent (non-equilibrative, concentrative) and sodium-independent (equilibrative) mechanisms but only the equilibrative transporter has been found in the vertebrate retina (Paes de Carvalho, 2002). Because equilibrative transporters are further subdivided as sensitive (IC_50_ in the nM range) or insensitive (IC_50_ > 1 μM) to NBTI, we used 10 μM NBTI to maximally and selectively block equilibrative transport.

NBTI (dissolved in dimethyl sulfoxide, 0.01% final) and all other drugs used in adenosine assay or electrophysiological recording experiments (dissolved in superfusion solution) were directly added to the superfusion solution. Control experiments indicated that 0.01% dimethyl sulfoxide did not affect the level of adenosine. Unless specified in the text, all compounds were purchased from Sigma–Aldrich (St. Louis, MO, USA).

#### Data Analysis

All data are expressed as the mean ± SEM of *n* values. With the assumption that data were normally distributed, the paired Student’s *t*-test was used (denoted with the symbol *) to compare two groups of paired values and the unpaired Student’s *t*-test was used (denoted with the symbol #) to compare two groups of unpaired values. To compare more than two groups of paired values, statistical analysis was performed using one-way repeated measurements analysis of variance (RM-ANOVA) followed by the Student–Newman–Keuls multiple comparison tests (Systat 11, Systat Software Inc., Richmond, CA, USA).

To test whether adenosine overflow varied with the time of collection in the long-term organotypic experiments, all measurements from an individual retina were averaged and then expressed as a fraction of the calculated mean. These relative values from different retinas were then averaged and plotted as a function of the time in culture. Statistical analysis of the values of adenosine overflow expressed as relative values to the average value of the rhythm was performed using RM-ANOVA (Ribelayga et al., 2004). Nonlinear regression analysis was then performed using SigmaPlot 9.0 (Systat Software Inc., Richmond, CA, USA). Individual values were fitted to the COSINOR equation (Nelson et al., 1979; Ribelayga et al., 2004):

y=M+Acos[2π(x−B)/τ]

where *y* is the *n*th data point (relative values), *x* the time of the *n*th data point (h), *M* the mean (mesor), *A* the amplitude (relative values), *B* the acrophase (radian) and *τ* the endogenous period (h). The regression coefficients are given with their respective asymptotic standard deviation estimates. The level for statistical significance of the regression coefficients was *P* < 0.001.

#### Spectral Sensitivity

Relative quantum sensitivity of L-type (H1) cHCs, a type of cHC that receives synaptic contact primarily from red (625 nm) cones (Stell and Lightfoot, 1975), was determined as described previously (Naka and Rushton, 1966; Wang and Mangel, 1996; Ribelayga et al., 2002, 2004). Data were normalized at the wavelength of peak sensitivity (550 or 600 nm). A 1 mV criterion response was used to minimize light sensitization of the dark-adapted state. “Light sensitization” refers to the phenomenon in which bright light (photopic range) stimulation of dark-adapted retinas increases the size of cHC light responses in the day and night (Mangel et al., 1994) and eliminates rod input to the cells during the night (Wang and Mangel, 1996). Rod spectral sensitivity data were obtained from Schwanzara (1967) and red (625 nm) cone spectral sensitivity data were obtained from Harosi and MacNichol (1974). The maximum, unattenuated light intensity of the stimulus at 550 nm was 7.2 × 10^12^ photons cm^−2^ s^−1^.

## Results

### Light/Dark and Day/Night Differences in Adenosine Levels in Freshly Isolated Goldfish Neural Retinas

Adenosine overflow (extracellular adenosine) and adenosine content (intracellular adenosine) of freshly isolated goldfish neural retinas were measured during the day and night of regular L/D and circadian cycles. Endogenous adenosine was detected at all times and under all illumination conditions. During a normal L/D cycle, both adenosine overflow and content were higher in darkness during the night compared to the day (Figures 3A,B). When a light background (white light in the low photopic range, 0.2 μW/cm^2^) was applied for 30 min following a 60 min period in the dark during day and night, adenosine overflow was significantly decreased in both day and night (Figure 3A). A 30-min period of light did not affect adenosine content during the day but decreased it by ~50% at night down to the daytime levels (Figure 3B). The day/night differences in the extracellular and intracellular levels of adenosine persisted under circadian conditions, that is when fish were kept in darkness for more than 24 h and sacrificed during the subjective day or night (Figures 3C,D). These observations demonstrate that endogenous levels of adenosine in the retina are regulated by light/dark adaptation and by a circadian clock.

Adenosine overflow was also detected from isolated goldfish retinal pigment epithelium + sclera (RPE-S) preparation, though no day/night differences were observed under dark-adapted conditions (*P* > 0.05, unpaired Student’s *t*-test). These observations indicate that the circadian rhythm of adenosine occurs in the neural retina and not the pigment epithelium.

### Extracellular Adenosine is Produced Extracellularly

To determine the source of extracellular adenosine, adenosine overflow was measured in the presence of the adenosine transport blocker NBTI (10 μM) and in the presence of NBTI plus the 5′-ectonucleotidase inhibitor, GMP (1 mM), which prevents the extracellular conversion of adenosine from AMP (which is itself converted from ATP extracellularly). Experiments were conducted during subjective day and night. As illustrated in Figure 4A, application of NBTI alone during the day increased adenosine overflow, a finding consistent with a flux of adenosine directed from the extracellular space toward the intracellular compartment. Additional application of GMP in the presence of NBTI decreased adenosine levels to an undetectable value, providing additional evidence for an extracellular origin of adenosine. Application of NBTI during the subjective night did not have any effect, though the application of NBTI + GMP dramatically affected adenosine overflow (Figure 4B). These findings are similar to those obtained in rabbit retina (Ribelayga and Mangel, 2005). Moreover, in the rabbit retina, we used two additional drugs to block the extracellular synthesis of adenosine. In particular, we used αβ-methylene adenosine diphosphate (αβmADP; 50 μM) to selectively inhibit 5′-ectonucleotidase, and 6-N,N-diethyl-D-β, γ-dibromomethylene-ATP (ARL67156; 50 μM) to selectively inhibit 5’-ectoATPase. In the presence of NBTI, the addition of GMP, αβmADP, or ARL 67156 suppressed retinal adenosine overflow, indicating that the source of extracellular adenosine is extracellular (Ribelayga and Mangel, 2005). Considered together, these observations suggest that extracellular adenosine is primarily produced extracellularly. Furthermore, the absence of an effect of NBTI at night suggests that equilibrative transport of adenosine is essentially absent at night.

### Circadian Oscillations of Adenosine Overflow From Explanted Fish Neural Retinas Persist for 2 Days

The finding that adenosine overflow was higher during the subjective night compared to the subjective day (Figure 3C) suggests that adenosine overflow is under the control of a circadian clock. To determine whether the clock is located in the retina, we maintained intact *in vitro* goldfish neural retinas in culture for 56 h in constant darkness (i.e., background illumination < −10 log *I*_o_) and temperature (20 ± 0.1°C), and measured the amount of endogenous adenosine that had accumulated in the culture medium every 4 h. Adenosine overflow was not constant over the course of 56 h and displayed day/night variations (RM-ANOVA, *F*_(12,48)_ = 5.852, *P* < 0.0001; Figure 5A). Cosinor analysis (see “Materials and Methods” section) further demonstrated that the extracellular level of endogenous adenosine exhibited a circadian rhythm (*r*^2^ = 0.93) with a period of 25.2 ± 1.6 h and an amplitude of 36 ± 4%. The finding that a circadian rhythm in adenosine release from *in vitro* retinas under constant darkness and temperature persisted for more than two full cycles demonstrates that the clock controlling the extracellular level of adenosine is located within the neural retina itself.

Because the RPE, which is in contact with the retina *in vivo*, releases ATP into the extracellular space (Pearson et al., 2005), adenosine overflow from the RPE-S was measured under the same constant dark and temperature conditions. In contrast to isolated neural retinas, no circadian variations of adenosine overflow were detected (RM ANOVA, *F*_(12,36)_ = 0.767, *P* = 0.669; Figure 5B). Rather, a constant level of adenosine from RPE-S was observed.

### Effects of Melatonin and Dopamine on Adenosine Overflow and Content

**Figure 6:**
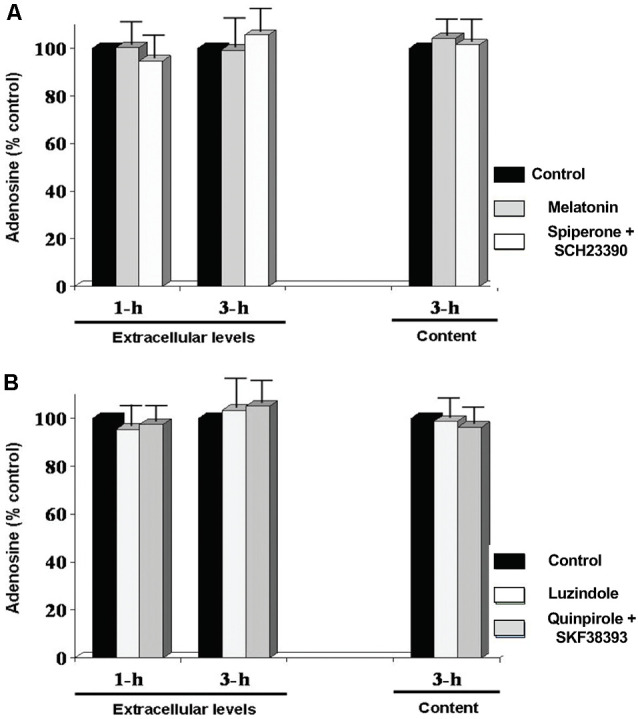
Circadian variations in retinal adenosine levels are not produced by the melatonin/dopamine system. Dark-adapted goldfish neural retinas were isolated in the middle of the day or night and superfused in darkness for 1 or 3 h. Subsequently, during the day **(A)**, melatonin (Mel; 100 nM), or a cocktail of the dopamine receptor antagonists spiperone (D_4_R) + SCH23390 (D_1_R) (Spip/SCH; 10 μM each) was applied for 30 min during continuous darkness. During the night **(B)**, the effects of the melatonin receptor antagonist luzindole (Luz; 10 μM) or a cocktail of the dopamine receptor agonists quinpirole (D_4_R) (Quin; 1 μM) + SKF38393 (D_1_R) (SKF; 10 μM) was tested under the same conditions. Results are expressed relative to the amount of adenosine measured after the initial 60 min-period in darkness (control). None of the treatments altered adenosine overflow or content. Data are from 5–6 retinas/condition ± SEM.

In several vertebrate species, including fish, melatonin has been identified as a direct output of a circadian clock expressed in the retina such that melatonin production and release peak at night (Iuvone et al., 2005; Mangel and Ribelayga, 2010; McMahon et al., 2014). Although the physiological impact of the rhythmic production of melatonin remains unclear in many species, we have previously shown that melatonin controls the circadian release of dopamine in the fish retina by inhibiting its release during the night so that dopamine release is low at night and peaks during the day (Ribelayga et al., 2004). We thus studied whether the rhythm of adenosine depends on melatonin and/or dopamine. To test this possibility, melatonin and dopamine receptor agonists and antagonists, which have been shown to alter the effects of the clock in goldfish retina (Ribelayga et al., 2002, 2004, 2008), were applied during the subjective day and night and adenosine overflow and content measured. Specifically, during the subjective day, which is when endogenous melatonin levels are the lowest and dopamine levels the highest, we applied melatonin (100 nM) or the dopamine receptor antagonists spiperone and SCH23390 (10 μM each), which block D_4_ and D_1_ receptors, respectively, for 1 and 3 h (Figure 6A). Conversely, following 1 h of darkness during the subjective night, which is when endogenous melatonin levels are high and dopamine levels are low, the D2R family dopamine receptor agonist quinpirole (1 μM) and the D_1_R agonist SKF38393 (10 μM) or the non-specific melatonin receptor antagonist luzindole (10 μM) were tested under the same conditions (Figure 6B). None of the tested drugs had an acute effect on adenosine overflow or content. These results suggest that the retinal circadian clock that controls the circadian rhythm of adenosine does not use melatonin or dopamine to mediate the daily rhythm in extracellular adenosine.

### Endogenous Activation of Adenosine A_2A_ Receptors Increases Rod-Cone Coupling at Night

Recent evidence suggests that activation of A_2A_Rs on cone terminals increases phosphorylation of connexin35 protein at the gap junctions between rods and cones (Li et al., 2009, 2013). However, it is not known whether endogenous activation of cone A_2A_Rs mediates the increase in rod-cone coupling at night. To test this, we injected Neurobiotin, a membrane-impermeable tracer molecule that diffuses through open (but not closed) gap junctions, into individual cones in intact neural goldfish retinas in the subjective day and night with or without A_2A_R ligands. We then measured the number of rods and cones to which the fluorescent tracer diffused in each condition.

**Figure 7:**
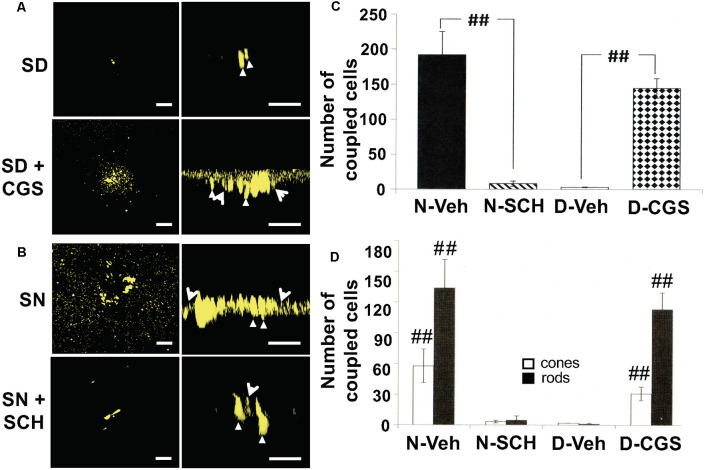
Circadian rhythm in rod-cone tracer coupling is mediated by endogenous activation of adenosine A_2A_ receptors. **(A,B)** Representative examples of the extent of Neurobiotin tracer diffusion through rod-cone gap junctions in the subjective day and night under four experimental conditions. **(A)** Only a few cells contained tracer after iontophoresis of neurobiotin during the subjective day whereas the presence of CGS21680 (CGS; 10 μM) during the subjective day increased the number of photoreceptor cells that contained tracer. **(B)** Tracer diffused into many cells during the subjective night (control) whereas the presence of SCH442416 (SCH; 0.5 μM) decreased the number of fluorescent cells. In each of the panels **(A,B)**, a confocal image of a whole-mount retina at the level of the rod inner segments is shown on the left, and a perpendicular view of the 3D reconstruction of the photoreceptor cells from the same retina is shown on the right. Some cones (arrowheads) and rods (arrows) are indicated in the panels on the right. In all cases, Neurobiotin was iontophoresed into individual cones in intact goldfish neural retinas (1 cone/retina) in an identical manner, i.e., by maintaining the recorded cells at +20 mV for 10 min. **(A,B)** Scale bars: 50 μm. **(C)** The Average number of stained photoreceptor cells following neurobiotin injections into individual cones in intact goldfish neural retinas (one cone/retina) under four dark-adapted experimental conditions. The number of tracer-coupled cells in the subjective night (control; *n* = 4, filled bar) was significantly greater (*p* < 0.01; unpaired Student’s *t*-test) than subjective night with SCH (0.5 μM; *n* = 4, striped bar). Also, the number of tracer stained cells in the subjective day in the presence of CGS (10 μM; *n* = 4, stippled bar) was significantly greater (*p* < 0.01; unpaired Student’s *t*-test) than subjective day control (*n* = 5, open bar). **(D)** The average number of stained cones (open bars) and rods (filled bars) (following Neurobiotin injections into individual cones (1 cone/retina) under the same experimental conditions as in **(A)**. The number of tracer-coupled cones and the number of tracer-coupled rods in the subjective night (control) were greater than the subjective night with SCH (0.5 μM; *p* < 0.01 for both rods and cones; unpaired Student’s *t*-test). Also, the number of tracer-coupled cones and the number of tracer-coupled rods in the subjective day in the presence of CGS (10 μM) were greater than the subjective day (control; *p* < 0.01 for both rods and cones; unpaired Student’s *t*-test). ^##^*p* < 0.01.

We found that under control conditions tracer was restricted to a few cells in the subjective day, indicating weak rod-cone coupling. In the presence of the general A_2_R (A_2A_R and A_2B_R) agonist 2-*p*-(2-Carboxyethyl)phenethylamino-5′-N-ethylcarboxamidoadenosine (CGS21680; 10 μM) during the subjective day the number of rods and cones containing tracer was significantly greater than observed during the subjective day under control conditions (Figures 7A,C,D), indicating strong rod-cone coupling. In contrast, in the presence of the selective A_2A_R antagonist SCH442416 (0.5 μM; Tocris, Minneapolis, MN, USA) during the subjective night the number of cells containing tracer was significantly lower compared to extensive diffusion of tracer into rods and cones in the subjective night under control condition (Figures 7B–D). These tracer coupling results, together with the finding that a circadian clock in the retina itself increases adenosine at night (Figures 3, 5), suggest that adenosine acts as a circadian clock signal for the night. The retinal clock increases endogenous A_2A_R activation at night, which increases the extent of rod-cone coupling.

In addition to measuring the extent of tracer coupling between photoreceptor cells, one can assess whether rod-cone gap junctions are functionally open or closed by determining whether rod input reaches cones (Ribelayga et al., 2008; Ribelayga and Mangel, 2010). Enzymatically isolated rods respond to 100-fold dimmer light stimuli than isolated cones. However, because cones in intact retinas receive rod signals through open rod-cone gap junctions at night, they respond to stimuli that are as dim as those to which isolated rods respond.

**Figure 8:**
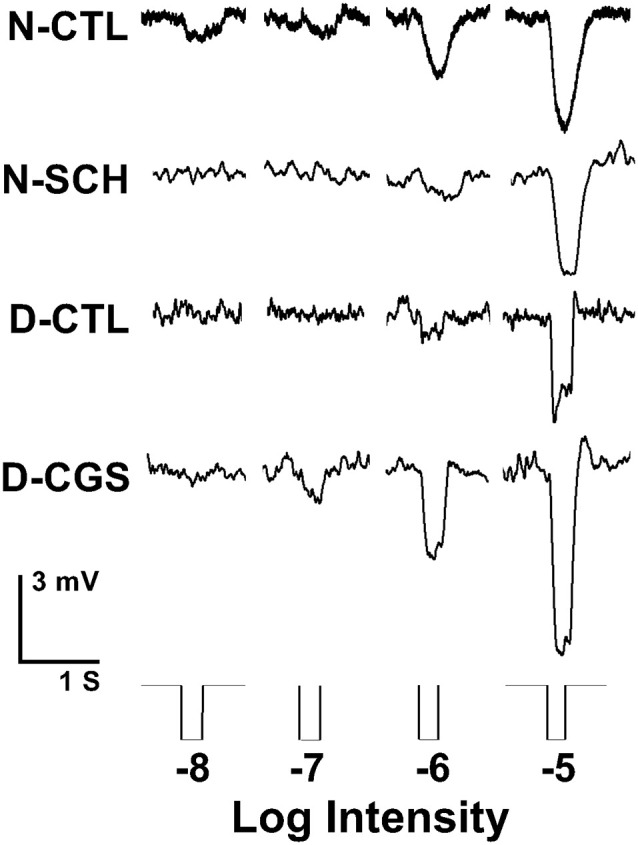
Endogenous activation of adenosine A_2A_ receptors increases rod input to cones at night. Representative examples of cone responses to a series of full-field white light stimuli of increasing intensity recorded during the subjective night, subjective night in the presence of SCH442416 (SCH, 0.5 μM), subjective day, and subjective day in the presence of CGS21680 (CGS, 10 μM) are shown. Cones responded to very dim light stimuli in the scotopic range during the subjective night (control) and in the subjective day in the presence of CGS, but not in the subjective day (control) and not in the subjective night in the presence of SCH.

**Figure 9:**
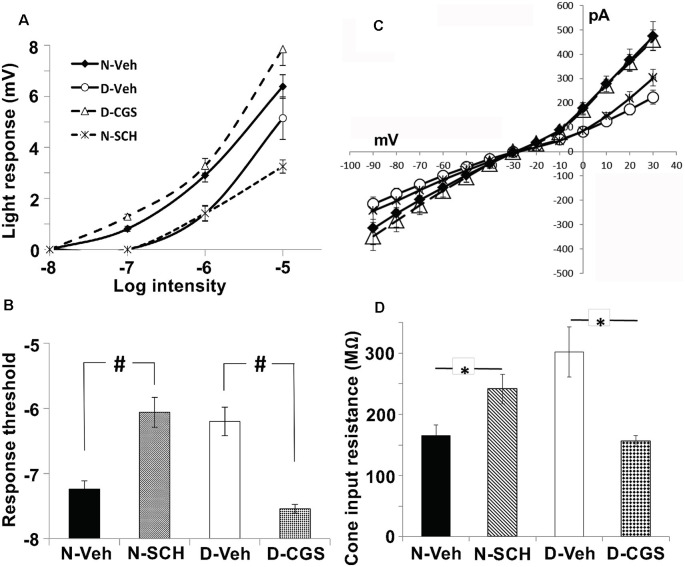
Endogenous activation of A_2A_Rs at night increases the sensitivity of cones to dim light stimuli and decreases cone input resistance. **(A,B)** Average intensity-response curves of cones **(A)** and average cone light response threshold **(B)** were measured during the subjective night (control; filled diamonds), subjective night in the presence of SCH (0.5 μM; stars), subjective day (open circles) and subjective day in the presence of CGS (10 μM; open triangles). **(A)** Average intensity-response curves of cones reveal that cones were more responsive at all intensities tested when recordings were obtained during the subjective night (control) and in the subjective day with CGS compared to the subjective day (control) and the subjective night in the presence of SCH. **(B)** Average cone light response threshold (intensity required to elicit a 0.5 mV response) was significantly lower in the subjective night (control) compared to the subjective night in the presence of SCH (*P* < 0.05; unpaired Student’s *t*-test), and in the subjective day with CGS compared to the subjective day (control; *P* < 0.05; unpaired Student’s *t*-test). All data points represent averaged responses to three stimuli at each intensity from 4–5 different retinas (1 cone/retina). **(C)** Relationship between peak membrane current (pA) and membrane (holding) potential (mV) of cones during the subjective night (control, N-Veh), subjective night in the presence of SCH (N-SCH), subjective day (control, D-Veh;), and subjective day in the presence of CGS (D-CGS) is shown. **(D)** Averaged cone input resistance was derived from these IV curves (one measurement/retina). The presence of SCH in the subjective night significantly increased (*P* < 0.05, unpaired Student’s *t*-test) cone input resistance (241 + 24 M-ohms, *n* = 6) compared to that measured in the subjective night (control; 166 + 17 M-ohms, *n* = 4). Also, the presence of CGS in the subjective day significantly decreased (*P* < 0.05, unpaired student’s *t*-test) cone input resistance (156 + 9 M-ohms, *n* = 4) compared to that measured in the subjective day (control; 302 + 41 M-ohms, *n* = 4). **(C,D)** The peak current was recorded when cones were voltage-clamped at −30 mV and stepped (duration 200 ms every 400 ms) from −90 mV to +30 mV in 10 mV increments. Input resistance calculations were restricted to holding potentials between −30 mV and −80 mV, the physiological voltage range for cones in which the relationship between voltage and current is relatively linear. ^#^*p* < 0.05; *p < 0.05.

We, therefore, performed whole-cell patch-clamp recordings of individual cones in intact goldfish retinas in the subjective day and night with or without A_2A_R ligands. On the subjective day, the average cone light response threshold was ~ −6.2 log *I*_0_ and responses were sharp and fast (Figures 8, 9A,B). In contrast, during the subjective night average cone light response threshold was ~ −7.2 log *I*_0_ (i.e., in the scotopic range, intensities to which isolated rods—but not isolated cones—respond), and light responses were significantly slower and longer in duration, indicating substantial rod input to cones (Ribelayga et al., 2008). Average cone light response threshold (~−6.0 log *I*_0_) and light response kinetics in the presence of SCH442416 (0.5 μM) during the subjective night resembled those observed during the subjective day (Figures 8, 9A,B), suggesting that endogenous activation of A_2A_Rs at night increases rod input to cones. Conversely, average cone light response threshold (~−7.5 log *I*_0_) and average light response kinetics in the presence of CGS21680 (10 μM) during the subjective day resembled those observed during the subjective night (Figures 8, 9A,B), suggesting that activation of A_2A_Rs increases rod input to cones and that A_2A_Rs are minimally activated during the day.

In the case of neurons that form gap junctions with neighboring cells, the overall input resistance reflects gap junction resistance in addition to membrane resistance. Thus, input resistance measurements can reveal changes in gap junction resistance. We, therefore, derived cone input resistance from I-V measurements (Figures 9C,D). Cones had lower input resistance in the subjective night (165.6 ± 17 MΩ) than in the subjective day (302.0 ± 41 MΩ, *p* < 0.05), a finding consistent with decreased photoreceptor gap junction resistance in the subjective night than subjective day. Moreover, average cone input resistance was significantly lower in the presence of CGS21680 (10 μM) during the subjective day (156 ± 9 MΩ, *p* < 0.05; unpaired student’s *t*-test) compared to the subjective day under control conditions. Also, average cone input resistance was significantly greater in the presence of SCH442416 (0.5 μM) during the subjective night (241 ± 24 MΩ, *p* < 0.05; unpaired student’s *t*-test) compared to the subjective night under control conditions. These results are consistent with the view that endogenous activation of A_2A_Rs at night increases the conductance of rod-cone gap junctions.

### Endogenous Activation of Adenosine A_2A_Rs at Night Increases Rod Input to Cone Horizontal Cells

If the retinal circadian clock uses adenosine to modulate rod-cone coupling and rod input to cones, then one should observe daily rhythms in rod input to cells post-synaptic to cones such as cHCs (Figure 1). By measuring responses to white and spectral light stimuli, previous work has shown that a circadian clock regulates rod input to cHCs in the goldfish retina by activating cone D_4_Rs (Wang and Mangel, 1996; Ribelayga et al., 2002, 2004). Specifically, the light responses of L-type cHCs during the subjective day are cone-mediated and similar to those previously reported (Naka and Rushton, 1966; Wang and Mangel, 1996). Conversely, the light responses of these horizontal cells are rod-dominated at night. As observed with rod horizontal cells, the responses at night are slower, smaller in size, and longer in duration, and the light response threshold is lower (Wang and Mangel, 1996).

**Figure 10:**
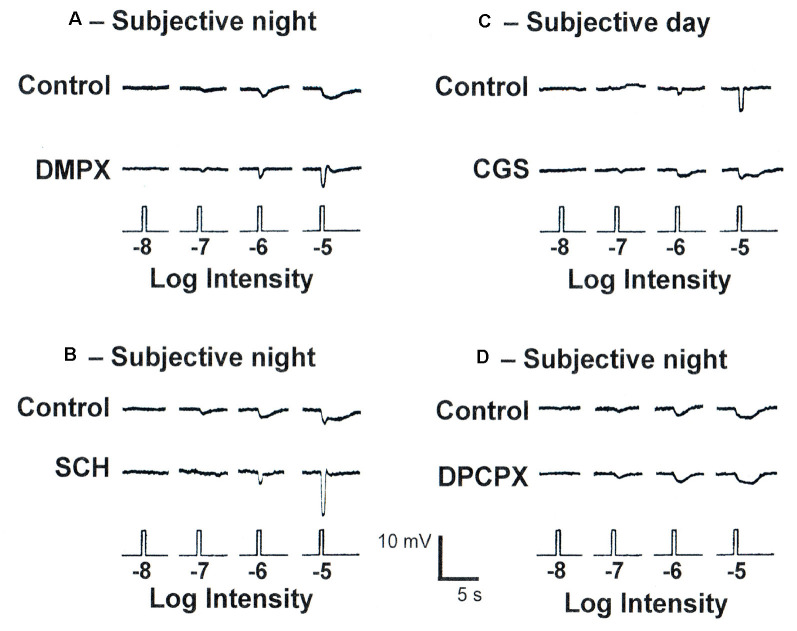
Endogenous activation of adenosine A_2A_Rs increases rod input and decreases cone input to goldfish retinal L-type cone horizontal (H1) cells at night. **(A,B)** Superfusion of DMPX (10 *μ*M) or SCH442416 (0.5 *μ*M) during the subjective night decreased rod input and increased cone input to L-type (H1) cHCs. No difference in the effects of DMPX and SCH442416 were observed. **(C)** Superfusion of CGS21680 (10 *μ*M) during the subjective day introduced rod input and decreased cone input to L-type cHCs so that light responses resembled those typically obtained during the subjective night. **(D)** Superfusion of DPCPX (10 *μ*M) during the subjective night had no effect. The recordings shown are representative of results obtained from between 6 and 9 cells in each experimental condition. In each case, control and test drug light responses were obtained from the same cell.

**Figure 11:**
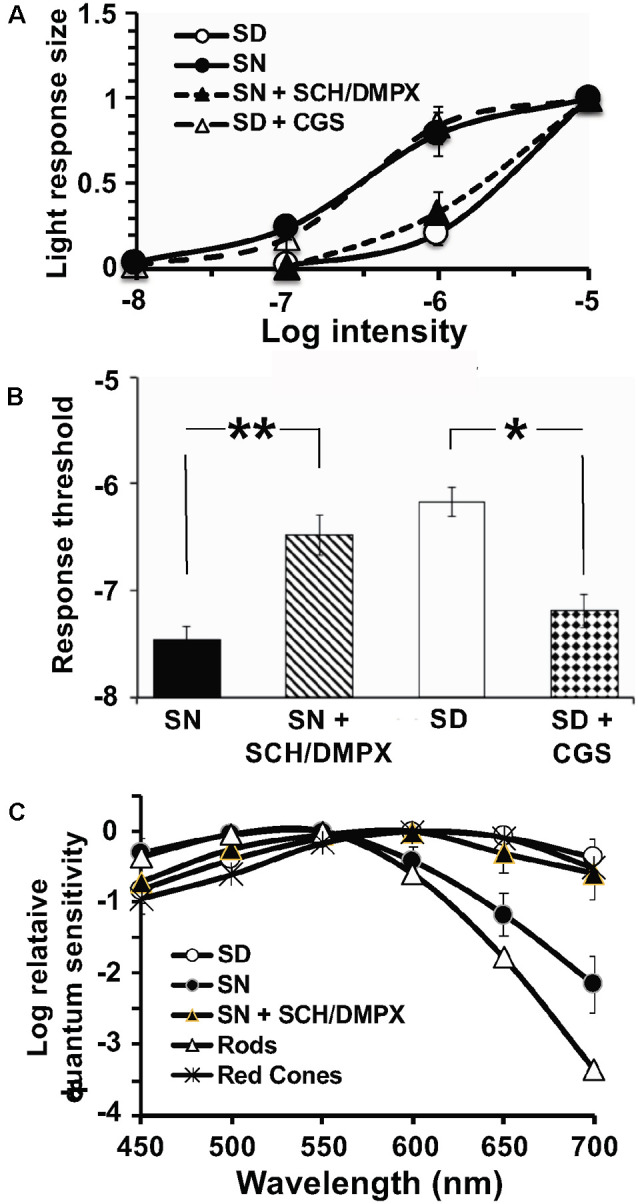
Endogenous activation of A_2A_Rs modulates light sensitivity and spectral sensitivity of L-type cHCs in the day and night. **(A,B)** Average normalized intensity-response curves of cHCs **(A)** and average cHC light response threshold **(B)** were recorded during the subjective night and subjective day before and after drug applications. Responses of each cell to light stimuli were measured in the subjective day and night both before and after drug applications. **(A)** Following superfusion of SCH or DMPX during the subjective night, average normalized intensity-response curves reveal that cHCs became less responsive to light stimuli at a range of intensities (−7 to −5 log *I*_o_) compared to before drug application. Also, the superfusion of CGS during the subjective day increased the responsiveness of the cells. Data in each experimental condition were normalized separately and averaged (see “Materials and Methods” section). All data points represent averaged normalized light responses from 6–9 different retinas (1 cell/retina). **(B)** Superfusion of SCH or DMPX during the subjective night significantly increased (*p* < 0.01; paired Student’s *t*-test) average light response threshold (intensity required to elicit a 0.5 mV response) compared to before drug application during the subjective night. Also, superfusion of CGS during the subjective day significantlydecreased (*p* < 0.05; paired Student’s *t*-test) the average response threshold. **(A,B)** SCH and DMPX data were pooled because no difference in their effects was observed. **(C)** The average spectral sensitivity of goldfish L-type cHCs resembled that of red (625 nm) cones (Harosi and MacNichol, 1974) during the subjective day, but resembled that of goldfish rods (Schwanzara, 1967) during the subjective night. Application of either SCH442416 (0.5 μM) or DMPX (10 μM) during the subjective night altered the average spectral sensitivity of L-type cHCs to resemble that of goldfish red (625 nm) cones, rather than goldfish rods. No difference in the effects of SCH442416 and DMPX was observed so the data were pooled. Each data point represents the average of 5–8 cells (1 cell per retina). *p < 0.05; **p < 0.01.

To determine whether the clock also uses adenosine to mediate its effects, fish cHCs were recorded during the subjective day and night and a series of pharmacological manipulations performed while measuring responses to white light stimuli of different dim intensities. Application of 3, 7-Dimethyl-1-(2-propynyl)xanthine (DMPX, 10 μM; Sigma–Aldrich), a general A_2_-like (A_2A_R and A_2B_R) receptor antagonist, or SCH442416 (0.5 μM) during the subjective night reduced rod input and increased cone input (Figures 10A,B, 11A,B) so that the responses resembled those typically observed in the subjective day. No difference in the effects of these antagonists was observed. Conversely, application of (CGS21680, 10 μM) during the subjective day increased rod input and decreased cone input to goldfish cHCs, a state typically observed during the subjective night in which the cells respond to very dim (i.e., low scotopic) stimuli (Figures 10C, 11A,B). Similar results were obtained when NBTI (10 μM) was added to the superfusate during the subjective day (data not shown). Also, the specific A_1_R antagonist 1,3-Dipropyl-8-cyclopentylxanthine (DPCPX, 10 μM) had no effect when applied during the subjective night (Figure 10D) or day (data not shown). These findings demonstrate that the retinal clock uses endogenous adenosine and adenosine A_2A_R activation to modulate rod and cone input to cHCs.

In addition to showing that cHCs respond to very dim (i.e., low scotopic) stimuli at night but not in the day, spectral sensitivity measurements have demonstrated that rod input to cHCs is modulated by endogenous activation of D_4_Rs (Wang and Mangel, 1996; Ribelayga et al., 2002, 2004). L-type (H1) cHCs, a sub-type of cHC, receive synaptic contact primarily from red (625 nm) cones (Stell and Lightfoot, 1975) and spectral sensitivity measurements during the subjective day (Figure 11C; Wang and Mangel, 1996; Ribelayga et al., 2002) support this. Figure 11C also shows that application of SCH442416 during the subjective night altered the average spectral sensitivity of L-type cHCs to resemble that of goldfish red cones (Harosi and MacNichol, 1974) and L-type cHCs during the day, rather than that of rods (Schwanzara, 1967).

The greater relative sensitivity of L-type cHCs to far red stimuli during the subjective night, compared to rods, has been reported previously (Wang and Mangel, 1996; Ribelayga et al., 2002) and may indicate that L-type cHCs receive some input from red cones at night, even though they are mainly driven by rods. A greater than expected sensitivity at night to far red stimuli was also observed in goldfish and Japanese quail electroretinogram studies (Barlow, 2001).

## Discussion

This study demonstrates that a circadian clock in the retina itself controls adenosine levels and activation of cone adenosine A_2A_Rs, which modulates neuronal light responses daily. More specifically, the results reported here show the following: (1) both extracellular and intracellular adenosine in the goldfish retina are modulated by a circadian clock and the background light level so that adenosine is highest at night in the dark (Figure 3); (2) the circadian clock that increases adenosine at night is located in the neural retina itself (Figure 5); (3) the circadian clock increases extracellular adenosine at night by increasing adenosine content so that inward transport of adenosine *via* equilibrative transport is stopped (Figure 4); (4) the level of extracellular adenosine is independent of melatonin and dopamine receptor activation (Figure 6) and dependent on enzymatic conversion of ATP into AMP and AMP into adenosine (Figure 4); and (5) the circadian clock- and darkness-induced increase in adenosine at night activates photoreceptor adenosine A_2A_Rs, which enhance rod-cone gap junction coupling (Figure 7) and rod input to cones (Figures 8, 9) and cHCs (Figures 10, 11). As a result, very dim light signals from rods reach cones and cHCs at night (but not in the day) due to endogenous activation of cone A_2A_Rs.

**Figure 12:**
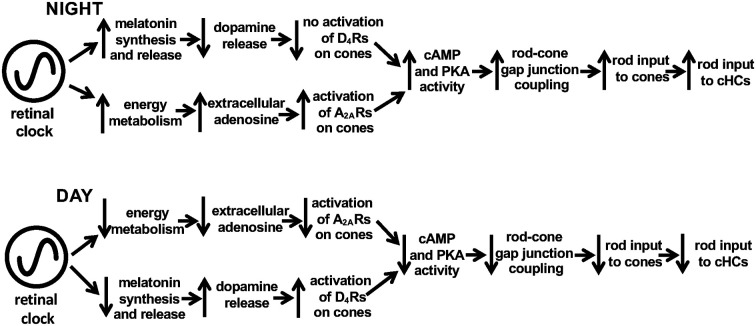
Schematic diagram showing adenosine- and melatonin/dopamine-mediated circadian clock pathways in the fish retina. Results in this study show that a circadian clock in the retina itself increases extracellular adenosine at night. The retinal clock is proposed to increase energy metabolism at night so that the extracellular level of adenosine increases. This in turn enhances activation of A_2A_Rs on rods and cones. As a result, intracellular cAMP and PKA activity levels in photoreceptors increase, thus enhancing the conductance of rod-cone gap junctions so that rod input to cones and then to cHCs is enhanced. Conversely, a clock-induced decrease in energy metabolism in the day lowers extracellular adenosine and A_2A_R activation. This lowers intracellular cAMP and PKA which closes rod-cone gap junctions so that rod input to cones and cHCs is decreased. This clock-controlled adenosine pathway is parallel to the clock-controlled melatonin/dopamine system. Previous work has shown that a retinal circadian clock increases melatonin synthesis and release during the night, which inhibits the release of dopamine from dopaminergic amacrine cells sufficiently so that D_4_Rs on photoreceptor cells are not activated. In contrast, the retinal clock decreases melatonin in the day, which enhances dopamine release, resulting in volume diffusion of dopamine throughout the retina and activation of D_4_Rs on rods and cones. This decreases intracellular cAMP and PKA activity levels in photoreceptors, which lowers the conductance of rod-cone gap junctions so that rod input to cones and cHCs is reduced. Note that separate circadian clocks may influence adenosine vs. melatonin/dopamine although one clock is depicted here controlling both pathways. See “Discussion” section for further details.

The increase in cone A_2A_R activation at night, which increases rod-cone coupling, plays a key role in producing the large day/night difference in the rod-cone coupling that has been observed (Ribelayga et al., 2008). This is because although rod-cone gap junctions are strongly uncoupled by the circadian clock-mediated increase in cone D_4_R activation in the day, cone D_4_Rs are NOT activated at night in the dark (Ribelayga et al., 2002, 2008), Thus, together with previous findings concerning circadian clock regulation of the melatonin/dopamine system (Mangel, 2001; Mangel and Ribelayga, 2010), the results reported here show that circadian clock activation of cone A_2A_Rs at night increases rod-cone coupling and rod input to cones and cHCs, while circadian activation of cone D_4_Rs in the day decreases rod-cone coupling and rod input to cones and cHCs (Figure 12). These opposite effects of the adenosine and melatonin/dopamine pathways enhance the day/night differences in rod-cone coupling and the sensitivity of cones and cHCs to very dim light stimuli, thus enhancing visual performance at night in a moonless environment and in the day when it is bright (see below).

Moreover, the results here establish that adenosine acts as an effector of the retinal clock. This conclusion was achieved by showing that circadian variations in adenosine persisted when the retina was explanted and cultured for several 24-h cycles in constant conditions (Figure 5). Electrophysiological and pharmacological experiments have further demonstrated a link between the circadian variation in adenosine and the day/night difference in the extent of rod-cone gap junction coupling (Figure 7) and rod input to cones and cHCs (Figures 8–11).

It is worth noting that the results reported here on goldfish retina can very likely be generalized across species including mammals because circadian pathway components and activity in the cone synapse are conserved across vertebrate species. For example, both A_2A_ and D_4_ receptors are located on cones in a variety of mammalian and non-mammalian retinas (Blazynski, 1990; Kvanta et al., 1997; Witkovsky, 2004; Li et al., 2013; Lohr et al., 2014; Dos Santos-Rodrigues et al., 2015), and a circadian clock in fish, rabbit and mouse retinas increases rod-cone coupling at night and decreases it in the day (Ribelayga et al., 2008; Ribelayga and Mangel, 2010).

### Mechanisms and Effects of Circadian Clock and Light/Dark Adaptive Control of Retinal Adenosine

The following observations in goldfish and rabbit retina support the conclusion that extracellular adenosine is produced extracellularly from the conversion of extracellular ATP into adenosine *via* the sequential actions of an ectoATPase and an ectonucleotidase and not from the intracellular conversion of AMP *via* the action of an endonucleotidase of extracellular origin: (1) ectoATPase and ectonucleotidase expression and activity are detected in the vertebrate retina (Braas et al., 1986; Blazynski, 1989; Blazynski et al., 1989; Paes de Carvalho et al., 1990); and (2) blockade of these enzymes dramatically decreases extracellular adenosine levels (Figure 4 here; Ribelayga and Mangel, 2005). Moreover, evidence indicates that ATP is present in the extracellular compartment and is released from Muller glial cells (Newman, 2015) and/or co-released synaptically with glutamate from photoreceptors (Dunwiddie and Masino, 2001; Latini and Pedata, 2001) or with GABA from dopaminergic amacrine cells (Ho et al., 2015). The synaptic origin of ATP is in agreement with the suppressive effect of light on adenosine overflow (Figure 3; Ribelayga and Mangel, 2005). Also, the fact that light did not totally inhibit adenosine overflow is in accord with a non-clock and non-light/dark regulated source of ATP that may originate from glial cells. We thus conclude that most extracellular adenosine in the retina originates from the extracellular conversion of ATP released from Muller glial cells, dopaminergic amacrine cells, and/or photoreceptor terminals. Also, we cannot exclude that *in vivo* a fraction of retinal extracellular ATP originates from retinal pigment epithelial cells (Pearson et al., 2005). Finally, because the release of ATP from dopaminergic amacrine cells can increase extracellular adenosine, it seems possible that dopaminergic amacrine cells may mediate both a light-adaptive signal *via* the release of dopamine and a dark-adaptive signal *via* the release of ATP and its subsequent extracellular conversion into adenosine.

In addition to the finding that extracellular adenosine is greater following maintained darkness than following maintained illumination, our results suggest a mechanism by which the retinal clock increases extracellular adenosine at night compared to the day. The level of extracellular adenosine reflects the rate of extracellular conversion of adenosine, the level of intracellular adenosine, and the rate and polarity of equilibrative adenosine transport. The findings that the equilibrative transport blocker, NBTI, had no effect during the subjective night but increased extracellular adenosine in the subjective day (Figure 4) suggest which one of three major possibilities is correct. First, the increase in intracellular adenosine content at night might be sufficient to reverse its inward flux so that it is transported outwards. We can rule out this possibility because if this were the case, we would have seen a decrease in extracellular adenosine in the presence of NBTI (Figure 4; Ribelayga and Mangel, 2005). Second, the clock might increase the extracellular production of adenosine at night. However, if this were so, we would have observed an increase in extracellular adenosine in the presence of NBTI (Figure 4). Third, the clock might lower adenosine reuptake activity at night. In fact, this is likely the case because NBTI did not have any effect on extracellular adenosine at night (Figure 4B; Ribelayga and Mangel, 2005), suggesting that transport activity was minimal. Because the clock increases intracellular adenosine content at night (Figure 3; Ribelayga and Mangel, 2005), it likely stops the inward flux of adenosine at night by increasing intracellular adenosine content and not by reducing transporter activity. Indeed, the latter possibility is consistent with a decrease in adenosine content at night. Therefore, considered together, our results suggest that the retinal circadian clock increases intracellular adenosine content at night sufficiently to stop its inward flux, so that adenosine accumulates in the extracellular space.

Because a circadian clock increases energy metabolism in both fish (Dmitriev and Mangel, 2000, 2004) and rabbit retina (Dmitriev and Mangel, 2001), likely, the clock-induced increase in the level of intracellular adenosine at night is due to a circadian-induced increase in energy metabolism (Figure 12). An attractive hypothesis is that neural activity and oxygen consumption may increase at night due to the action of the clock so that a slightly hypoxic condition is generated, thereby triggering the intracellular accumulation of AMP, a substrate for adenosine. In support of this, anoxic and hypoxic experimental conditions increase adenosine content and overflow in rabbit retinas (Ribelayga and Mangel, 2005).

It is important to note that a limitation of our technique is that we measure global overflow and the content of endogenous adenosine, and this may likely underestimate local variations in adenosine concerning specific retinal layers. Although the retinal adenosine system is well conserved among vertebrates, it is not homogeneously expressed in different retinal layers (Blazynski and Perez, 1991). For instance, 5′-nucleotidase activity is detectable mainly in the outer retina, whereas adenosine deaminase is expressed in the inner layers of the retina, where adenosine immunoreactivity is mostly found (Braas et al., 1986; Blazynski, 1989; Blazynski et al., 1989; Paes de Carvalho et al., 1990). Also, [^3^H]-adenosine reuptake occurs mainly in the inner nuclear and ganglion cell layers (Blazynski et al., 1989; Blazynski and Perez, 1991). In goldfish, rod horizontal cells represent the main cell-type that takes up radiolabelled adenosine (Studholme and Yazulla, 1997). Taken together, these data suggest that the inner retina acts as a sink that scavenges adenosine produced extracellularly in the outer retina. Yet, despite these limitations, our direct measurements of endogenous adenosine confirm the presence of a functional adenosine system in the vertebrate retina and establish that it is controlled by both the background light level and a circadian clock located in the neural retina itself.

### On the Relationship Between the Retinal Clock, Dopamine, Melatonin, and Adenosine

Although melatonin and dopamine have been characterized as major outputs of the vertebrate retinal clock (Mangel, 2001; Iuvone et al., 2005; Besharse and McMahon, 2016), our results suggest that the circadian variation in the level of adenosine is independent of endogenous melatonin and dopamine receptor activation and the circadian rhythms in melatonin and dopamine. These observations further suggest either that a single retinal clock has two distinct outputs (Figure 12) or that there are two distinct circadian clocks in the vertebrate retina, one controlling melatonin signaling and the other controlling adenosine signaling. Given the widespread expression of circadian clock components in the retina (McMahon et al., 2014), either possibility might be correct. However, evidence suggests that rhythmic melatonin synthesis occurs primarily within the photoreceptor layer whereas adenosine accumulates in cells of the inner retina. This difference suggests that the two circadian pathways may indeed rely on separate clocks located in different retinal layers. Recent research has demonstrated that each retinal layer contains functional circadian clocks (Jaeger et al., 2015) but the specific clock cell types that control melatonin and adenosine levels remain to be identified.

Whereas manipulating melatonin or dopamine signaling did not significantly affect adenosine overflow or content (Figure 6), previous studies have reported a facilitating effect of adenosine on melatonin production in the vertebrate retina (Haque et al., 2003; Ivanova and Iuvone, 2003a,b), as well as an inhibitory effect on dopamine release (Michaelis et al., 1988; Crosson et al., 1994). The nighttime increase in extracellular adenosine may therefore reinforce the effects of the clock on the melatonin and dopamine systems at night. Thus endogenous adenosine may augment the day/night difference in rod-cone coupling by increasing coupling at night in two distinct ways: (1) by directly activating A_2A_Rs on cones, as described in this study; and (2) by potentiating the action of the clock on melatonin production to synergistically inhibit dopamine release.

### Physiological Consequences of the Clock-Controlled Adenosine Rhythm

The A_2A_R-mediated increase in the coupling between photoreceptors at night enhances the signal-to-noise ratio and the reliability of rod responses to dim visual stimuli (Lamb and Simon, 1976), especially to large dim objects (Ribelayga et al., 2008; Mangel and Ribelayga, 2010; Ribelayga and Mangel, 2010; Jin et al., 2015). Also, increased photoreceptor coupling may improve contrast detection and detection of small spatial details (Lebedev et al., 1998). Moreover, the opposite effects of endogenous activation of cone A_2A_Rs and D_4_Rs in modulating rod-cone coupling may facilitate the switch between rod and cone circuit function at dusk and dawn.

Our observations of the role of adenosine in the circadian clock and dark-adaptive processes in the outer retina are consistent with previously reported effects of exogenous adenosine on the biochemistry and physiology of the outer retina. These include the facilitating effects of adenosine on cone myoid elongation in the fish retina (Rey and Burnside, 1999) and on melatonin synthesis in chicken (Haque et al., 2003), processes known to occur at night under the control of a circadian clock. Adenosine has also been reported to have a variety of inhibitory effects. In the tiger salamander, adenosine inhibits rod opsin mRNA expression (Alfinito et al., 2002), calcium influx through L-type calcium channels in rods (Stella et al., 2002), and cones (Barnes and Hille, 1989), suggesting that adenosine inhibits glutamate release. Adenosine is also a competitive inhibitor of rhodopsin kinase, and thus inhibits rhodopsin phosphorylation (Donner and Hemilä, 1985; Palczewski et al., 1988).

In summary, a circadian clock in the vertebrate retina itself controls endogenous adenosine levels and A_2A_R activation in a manner that is independent of melatonin and dopamine receptor activation. A clock-mediated increase in A_2A_R activation at night augments rod-cone coupling and rod input to cones and cHCs. These effects, together with the effects of a separate melatonin/dopamine clock system in the retina that strongly decreases rod-cone coupling in the day, act in concert to enhance the day/night difference in rod-cone coupling. Clock control of rod and cone pathways in the outer retina thus facilitates detection of large, very dim objects at night and fine spatial details in the day, enhancing the ability of animals to survive as the visual environment changes daily.

## Data Availability Statement

The raw data supporting the conclusions of this article will be made available by the authors, without undue reservation.

## Ethics Statement

The animal study was reviewed and approved by the Institutional Animal Care and Use Committee at The Ohio State University, Columbus, OH, USA.

## Author Contributions

JC, CPR, and SCM designed the study, collected experimental data, performed statistical analyses, and wrote the manuscript. All authors contributed to the article and approved the submitted version.

## Conflict of Interest

The authors declare that the research was conducted in the absence of any commercial or financial relationships that could be construed as a potential conflict of interest.
